# Discussion of development processes in insect‐fungus association derived from the shaggy parasol fruiting on the nests of hairy wood ants

**DOI:** 10.1002/ece3.5611

**Published:** 2019-09-30

**Authors:** Douglas Fraser

**Affiliations:** ^1^ Department of the Natural and Built Environment Sheffield Hallam University Sheffield UK

**Keywords:** ant‐fungus association, *Chlorophyllum rachodes*, *Chlorophyllum rhacodes*, *Formica lugubris*, fruit‐body distribution, fungus cultivation evolution, *Macrolepiota rhacodes*, rhizosphere ecology, wood ant nests

## Abstract

1. *Chlorophyllum rhacodes*, typically regarded as a rich grassland or open forest “mushroom” species, was found fruiting abundantly on nests of *Formica lugubris*, occurring in a *Pinus silvestris* plantation. Fruiting was absent from the rest of the woodland.
2. Research focussed on the activities in the nests that could explain this. Within nests, there was a spatial relationship between *C. rhacodes* mycelium, insect cadavers, fruitbody initiation, and roots of adjacent trees.
3. In vitro experiments found that *C. rhacodes* was not mycorrhizal with *P. silvestris*, but that it had qualities which rendered it suitable for colonization of the rhizosphere in the conditions of the nest mound and for further niche development.
4. Implications of the unusual presence of fruit‐bodies and the distribution of associated hyphae are discussed in relation to the nutritional biology (and recent taxonomical reassignment) of the fungus. This includes reference to the relevant physiology of insects and to the accepted evolution of mutualistic symbioses between fungi and the Attini and Termitidae.
5. An argument is presented that the situation observed in vivo provides evidence of a degree of facultative association and what could be tangible support for the theory for the developmental origin of mutualistic fungus cultivation by insects. It is presented as a context for continued experimental research.

## Graphical abstract

*Chlorophyllum rhacodes*, an open forest 'mushroom' species, is occasionally found fruiting on nests of *Formica lugubris*. Within less mobile thatch of such nests, there was a spatial relationship between *C. rhacodes* mycelium, insect cadavers, fruitbody initiation, and roots of adjacent trees. The situation provides evidence of a degree of facultative association in the rhizosphere. A discussion is presented for a potential developmental mechanism in support of the accepted theory for the developmental origin of mutualistic fungus cultivation by insects.

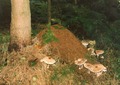

## 1 INTRODUCTION

### 1.1 Rationale

This paper describes an interdisciplinary, natural fungus‐insect‐rhizosphere interaction and presents both experimental attempts and discursive theory to elucidate some ecological processes and explanations. The discussions present a view on the development of:

1. *Chlorophyllum rhacodes* as an inhabitant of ant nest mounds: habitat requirements (physical and nutritional, including specific enzymatic capabilities of the fungal tribe) and potential for associations.
2. The potential effects of mycophagy in *Formica lugubris* in the light of mutualistic development models.

### 1.2 The observation

*Chlorophyllum rhacodes* (Vittadini) Vellinga (formerly *Macrolepiota rhacodes* (Vittadini) Singer, a.k.a. shaggy or reddening parasol mushroom) is an open woodland litter saprotroph (associated more with conifer than broadleaved woodlands) and is possibly found in grasslands (Legon & Henrici, 2019; Vellinga, 2004; Žižka & Gabriel, 2008). Over a four‐year period, fruit‐bodies of *C. rhacodes* were observed, by the author, on the nest mounds of the northern or hairy red wood ant, *Formica lugubris* Zetterstedt (Hymenoptera: Formicidae), in Highlow Wood, a coniferous plantation in the Peak District National Park, UK. No fruit‐bodies of this fungus were seen in surrounding soils or any other substratum in the woodland. Popular guides have noted it in the United States (Savonius, 1973); it has been described generally as occurring on ant hills (Arora 1986; Dickinson & Lucas, 1982), and more specifically in Washington State (Stamets, 1990, pers. com.). It has been recorded fruiting on old wood ant nests at the same location, in the UK, in 1986, and in other cooler areas of North and West Europe, as well as forming 'fairy rings' on poultry pasture (with mature oaks) in Oregon, USA (Sagara, 1992), and on rich soils derived from compost piles, mulch, farmyards, stable litter (Arora 1986; Dickinson & Lucas, 1982). Sagara's (1992) review suggests a saprotrophic fungus with a habitat selectivity for stable, nutritionally rich, humic substrata. Kilpeläinen's et al. (2008) review of ant nests suggests that such conditions are found in abandoned mounds where the structural materials begin to rot.

Highlow Wood (compartment H8b) was a plantation of (predominantly) fifty‐year‐old *Pinus silvestris* L. (Scots pine) with some *Larix decidua* Mill. (European larch). Within the Peak District National Park, UK, the site is situated in a sheltered valley, orientated approximately east–west, with Highlow Brook, a narrow tributary of the River Derwent running through it. Location: centered on O.S. grid ref: SK224799; Latitude: 53°18′54″N, Longitude: 1°39′53″W; elevation: 180 m AOD.

## 2 MATERIALS AND METHODS

### 2.1 Fruiting period and distribution

Field observations were carried out over a period of two years in Highlow Wood. Nest mounds of *F. lugubris* were mapped, and fruiting of *C. rhacodes* was noted for position on nest mounds and duration. Nest conditions (temperature, pH and structure) were mapped against the presence of *C. rhacodes* fruit‐bodies. Nest mounds were dissected to follow mycelial cords through the nest structure.

### 2.2 Habitat requirements of *C. rhacodes*

Laboratory tests were carried out for the ability of *C. rhacodes* to form a mycorrhiza. The first method used was developed by R. Bradley and D.J. Read (pers. com.) with medium pH amended to match average nest conditions (pH 4.7), and subsequently with potted seedlings in previously sterilized compost, inoculated with mycelial mats from the base of *C. rhacodes* fruit‐bodies. Roots were observed macroscopically for the characteristic shape of ectomycorrhizal development. Microscopic observations were carried out on roots sectioned and stained with methyl blue to highlight fungal mycelium.

The ability of *C. rhacodes* to utilize ants or their (imago) prey cadavers was investigated. Specifically, lipolytic and protease activities were tested, as was the ability to degrade chitin. *C. rhacodes* was grown on the indicator medium for lipolytic activity (Sierra, 1957) and on milk agars and chitin substrates. Lipolytic activity would be recognized by the appearance of crystalline precipitate of insoluble fatty acids. The production of exogenous proteases would clear milk agars. The ability to metabolize purified (white) chitin was measured by assaying the amount of N‐acetylglucosamine (released by depolymerization of chitin) after growth in a chitin‐rich agar medium.

### 2.3 Potential for consumption of *C. rhacodes* by *F. lugubris*

Freeze‐dried specimens of *F. lugubris* (from the investigation site) were examined under scanning electron microscope (SEM) for any recognizable adaptations for mycophagy. Ant fecal pellets were examined for presence of passaged spores.

## 3 RESULTS

### 3.1 Fruiting period and distribution

*Chlorophyllum rhacodes* fruiting on the nests began in May and continued well into December. Within a season, there were normally multiple flushes recorded on the nests (maximum of five flushes, totaling 29 fruit‐bodies on the same area of a nest).

In this study, temperatures recorded throughout the nests were generally uniform; slightly higher than still ambient air, and as much as 22°C higher than surrounding soil. This could allow a longer season of vigorous mycelial growth and may explain the protracted fruiting season.

*Chlorophyllum rhacodes* fruit‐bodies reached maturity, in an unmolested state, only on active ant nest mounds. On recently abandoned ant nests, fruiting was initiated, but the success of fruit‐body maturation was always zero, with primordia always being fatally damaged by fungivorous activity. This suggests that, in this area, fruiting success appeared to be linked, spatially and temporally, to ant nest activity.

Of the 12 nests present at the start of the study, only six produced fruit‐bodies every season. All of the latter were inhabited throughout this period and all were built at the bases of living pine trees.

**Figure 1:**
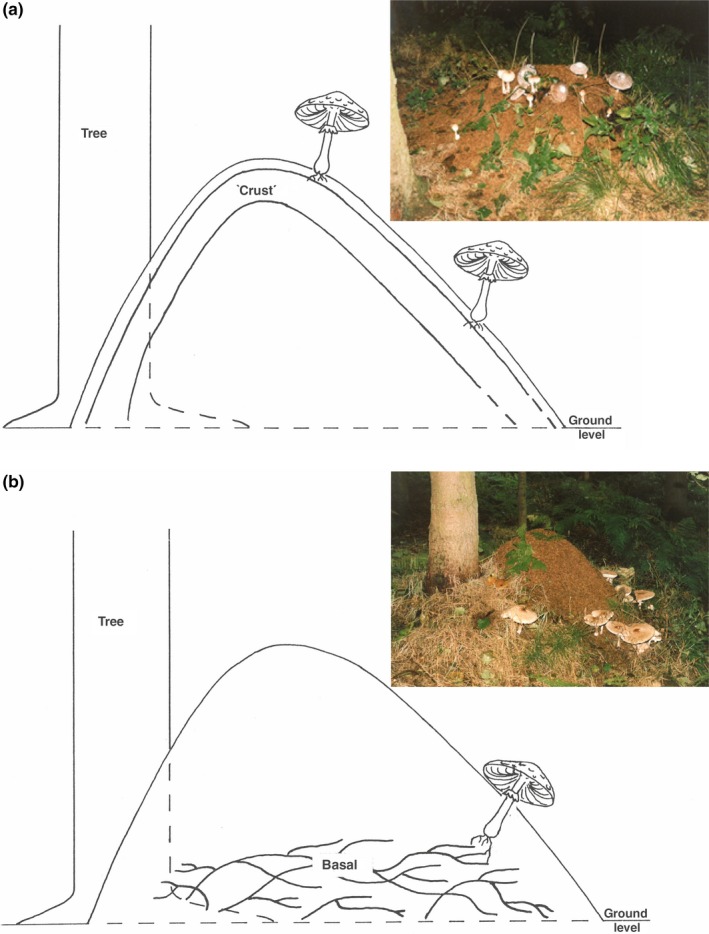
(a) The position of the bulk of tree roots in a nest exhibiting a ‘crust’ distribution and associated ‘blanket’ distribution of *Chlorophyllum rhacodes* fruit‐bodies. Inset photograph demonstrates ‘blanket’ distribution and bulbous bases of stipes are at the surface, indicating fruitbody initiation near the surface (and ‘crust’). (b) The position of the bulk of tree roots in a nest exhibiting a ‘basal’ distribution and giving rise to a ‘peripheral’ distribution of *Chlorophyllum rhacodes* fruit‐bodies, which are also demonstrated in the inset photograph

**Figure 2:**
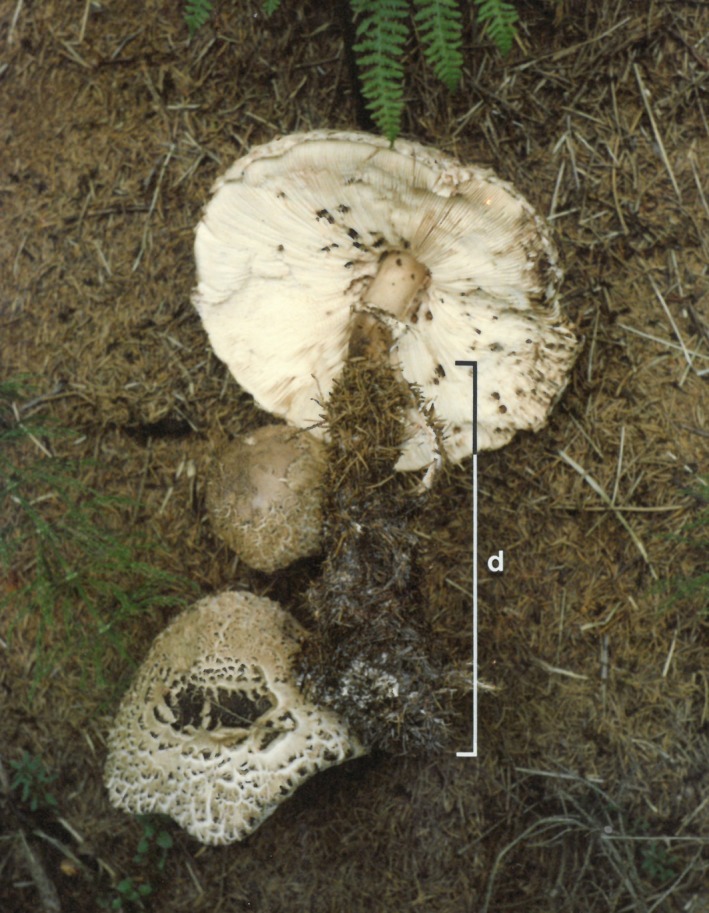
‘Peripheral’ fruiting was often associated with long, subterranean stipe lengths (d), which originated near or in the ‘basal’ root mat. The maximal length of d may have prevented fruit‐bodies from appearing higher up the crown of the nest

Excavation of the nests revealed that there was an obvious physical association between the tree roots and dense, white mycelium, and fruit‐body primordia initiation. Fruit‐bodies were found in two patterns of distribution on the nest mounds, and these patterns were linked to specific patterns of tree root invasion of the nests. Roots from the adjacent trees were found in either a basal pattern or a crust pattern (Figures 1a,b). Where roots were basal, fruit‐bodies were distributed in a peripheral pattern, around the lower third of the nest height. Where roots were distributed in a basal pattern, fruit‐bodies occurred in a blanket distribution, spread over the entire crown of the nest (Figure 1b). Tree root mass was often covered with an obvious (white) mycelium. Mycelial cords emanated from the root‐hypha mats and were easily traced to the base of *C. rhacodes* fruitbody stipes. In that, the cords were never longer than 5 cm, the fruit‐bodies were initiated close to the roots. This close physical association leads to the distribution of fruit‐bodies (crust roots with blanket fruit‐bodies and basal roots with peripheral fruit‐bodies) where the maximum length of stipe limits how far up the nest the fruit‐body appears (Figure 2).

In nests with a crust distribution of roots, the rhizosphere could be very close to what appeared to be galleries containing complete (nonant) insect cadavers. Food store galleries in *Formica* spp. nest mounds are not recorded in the literature. These apparent “galleries” may be the result of remains accumulating in the relatively immobile (rhizosphere) area of the nest mound. Hyphal cords associated with the roots extended to the fruit‐body stipes. They were also associated with cadavers, on which they split into individual hyphae. There was a noticeable hyphal load in and on these cadavers.

### 3.2 Habitat requirements of *C. rhacodes*

#### 3.2.1 Test for ectomycorrhizal relationship between *C. rhacodes* and *P. sylvestris*

The propinquity of mycelium and roots suggests an association. Stubby, dichotomous root branching suggested an ectomycorrhiza. Microscopy revealed evidence of a Hartig net on roots, but when *C. rhacodes* was tested for its ability to form mycorrhizal association with *P. silvestris*, no mycorrhizal roots were developed. Indeed many roots sustained cortical damage, which led to root die‐back. The fungus did not thrive in the axenic, moist environment, so the investigation was attempted in plant pots. A strong ectomycorrhiza was not developed, but there were several instances of hyphae penetrating between healthy root cortical cells.

#### 3.2.2 Relationship with conditions provided by *F. lugubris*

**Figure 3:**
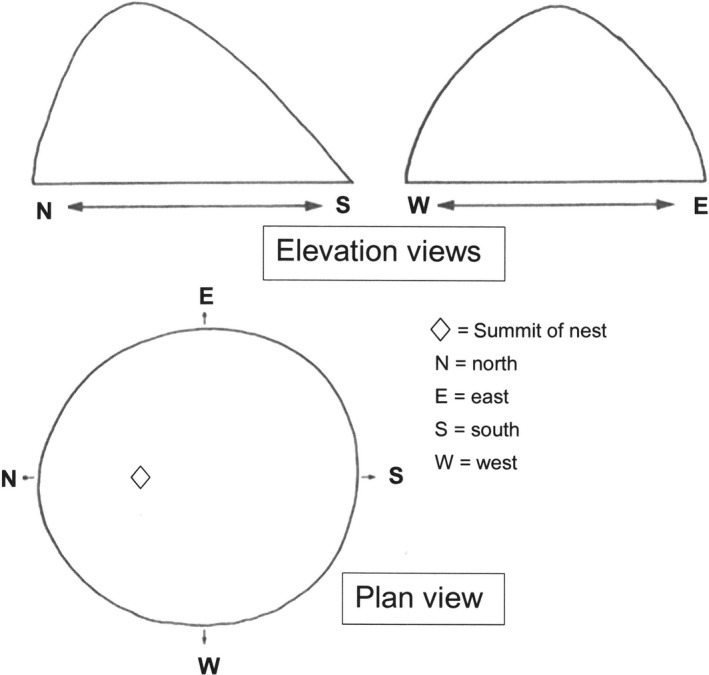
The asymmetry of the nest mound exposes a greater surface area to the warming influence of the sun

Temperatures recorded throughout the nests were generally uniform; slightly higher than still ambient air, and up to 22°C higher than soil surrounding the nests. This could allow a longer season of vigorous mycelial growth and may explain the protracted fruiting season. Nests are sited most frequently where they benefit from passive solar gain (as Kilpeläinen et al., 2008; Robinson, Tofilski, & Ratnieks, 2008). This is near the linear gap in the canopy, formed by the river, especially on the south‐facing aspect of Highlow Wood. It is most likely that the ants are using insolation to increase nest temperature, which is further regulated by creating or blocking openings in the nest mound. In the relative shade of the woodland, the shape of the nest mound is often asymmetric, exposing a greater surface area at an angle approaching 90° to the sun's rays (Figure 3). When areas were clear‐felled, the nest shape, after recovery, was more symmetric and less high.

#### 3.2.3 Relationship with resources provided by *F. lugubris*

##### Nutrient (ammonium nitrogen) availability in rhizosphere area of nest mounds

Nests are composed of litter and the frequent redistribution of the material maintains an environment suited to the ants and avoids rapid decomposition. The mixture of tree roots, nest material, and hyphae presented a combined mass that might be less likely to be moved by ants in their general nest material rotation. Kilpeläinen's et al. (2008) study of the nests of six species of *Formica* ants suggested that the rotation (which slows the degradation of materials), therefore, held nutrient resources in the nests until the ant activity ceased on abandonment of the nests. Thus, the same argument suggests that in the area of the nest consolidated by tree roots and hyphae, degradation processes are not being prevented and release of nutrients is happening. The rhizosphere area of the nest mound probably experiences more release of saprophyte‐suitable nutrients than other parts of the nest mound. Root exudates contain sugars that stimulate microbial growth, which, in turn will enhance the decomposition of the immediately surrounding litter material of which the nest mound is built. This will lead to a release of ammonium (soluble) nitrogen.

To assay total ammonium nitrogen of different regions, nest crowns and adjacent soils were divided into approximately 20 × 10 × 10 cm portions. Preparation involved thorough mixing of each sample and removal of all recognizable, living plant material.

**Figure 4:**
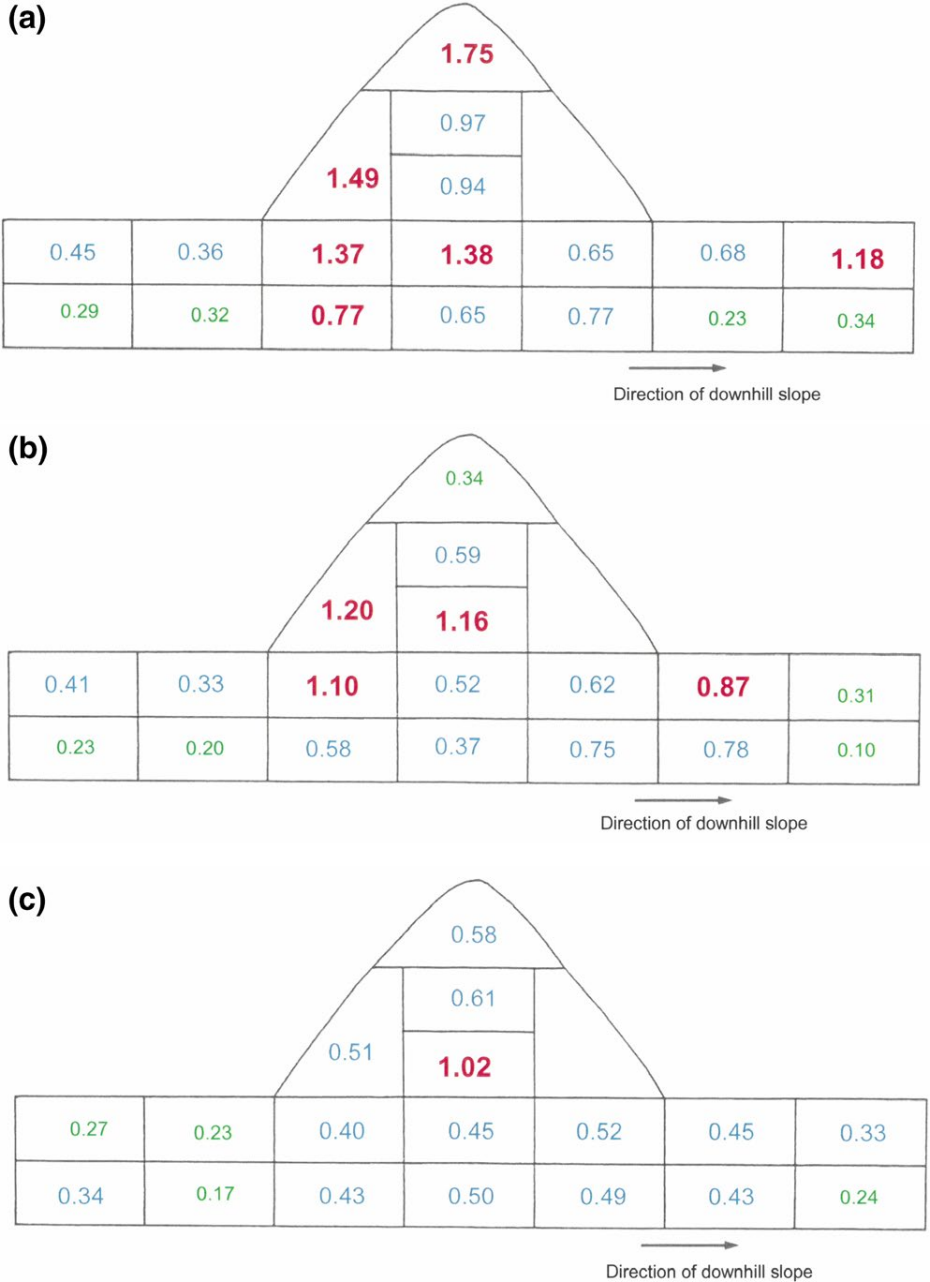
(a) Ammonium nitrogen content (mgg^−1^ dried sample) of different areas of an active nest exhibiting ‘crust’ distribution of tree roots and ‘blanket’ production of fruit‐bodies. Type size and color change indicate a difference at *p* = .05. (b) Ammonium nitrogen content (mgg^−1^ dried sample) of different areas of an active nest exhibiting ‘basal’ distribution of tree roots and ‘peripheral’ production of fruit‐bodies. Type size and color change indicate a difference at *p* = .05. (c) Ammonium nitrogen content (mgg^−1^ dried sample) of different areas of an active nest with no fruit‐body production and no tree root penetration. Type size and color change indicate a difference at *p* = .05

The highest nitrogen levels were found in the nests on which fruit‐bodies were observed. Within these nests, the highest nitrogen levels were recorded in the rhizosphere areas, where fruit‐bodies were initiated (Figure 4a–c).

##### Cellulytic, lipolytic, and protease activity in *C. rhacodes*

The lepiotaceous fungi (including *C. rhacodes*) normally utilize fast‐decomposing litter, utilizing cellulose, hemicellulose, and lignins (Vellinga, 2004; Valášková et al. 2007 *cited in* Žižka & Gabriel, 2008). However, the related fungus, *Leucocoprinus gongylophorus*, associated with attine ants, derives polysaccharides from sources other than cellulose because of its interaction with the ant nest community (Bacci, Anversa, & Pagnocca, 1995). The distribution of visually traceable *C. rhacodes* mycelium was associated with roots and insect cadavers, suggesting alternative sources of nutrition to litter alone. So the following considerations were tested.

The rhizosphere 'galleries' contained large numbers of insect cadavers (including soft‐bodied larvae and instars and imagos with harder exoskeletons) and, therefore, present a source of fatty acids and proteins. To investigate if *C. rhacodes* can utilize these resources, it was grown on the indicator medium for lipolytic activity (Sierra, 1957) and on milk agars and chitin substrates.

Lipolytic activity would be recognized by the appearance of crystalline precipitate of insoluble fatty acids. No such evidence was found.

**Figure 5:**
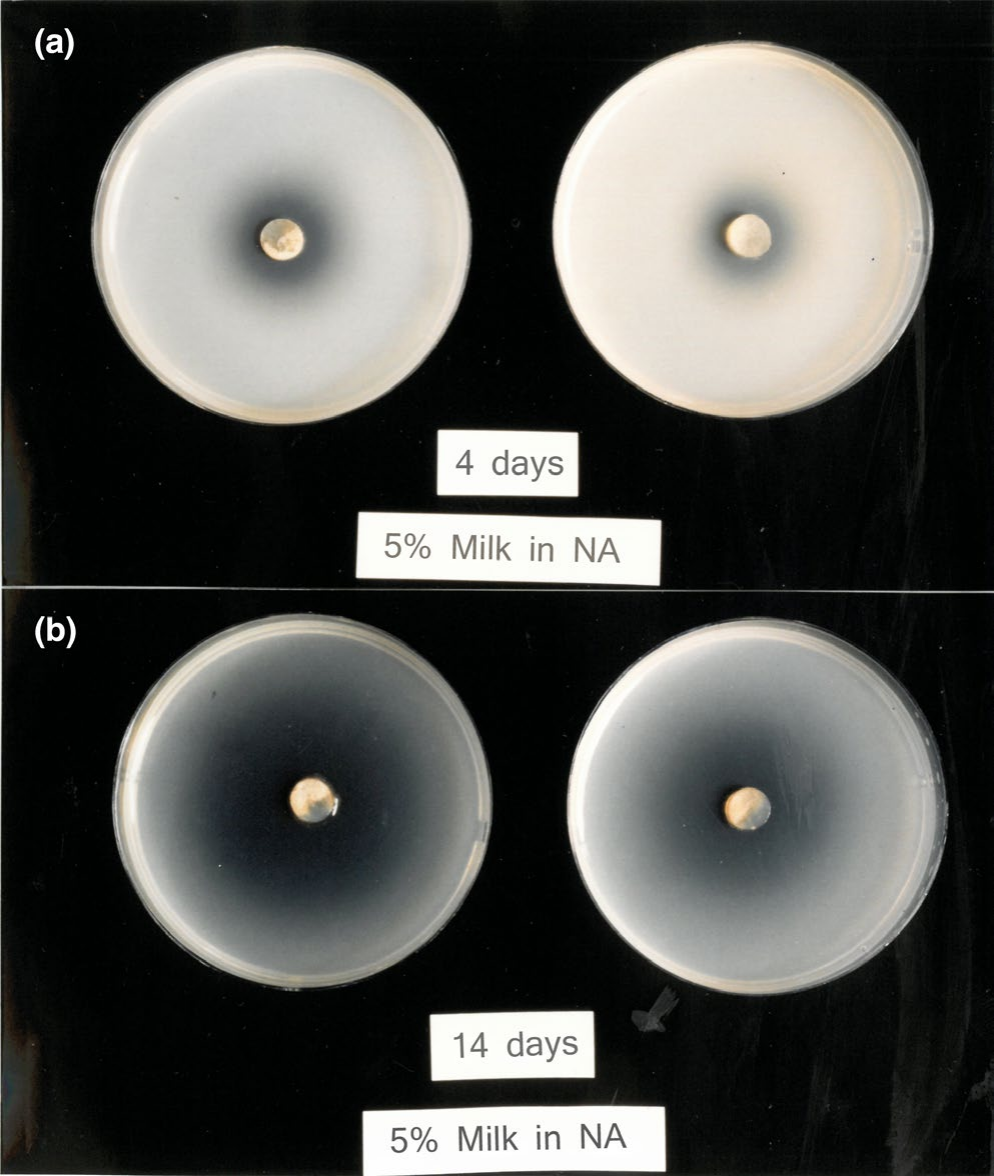
Milk nutrient agar was inoculated with *Chlorophyllum rhacodes*. Substantial enzyme activity is demonstrated by clearing in the agar (shown against a dark background), after 4 and 14 days. Caution is advised in interpretation, however, as this medium does not support growth of this fungus, so results may be enhanced by endogenous proteases leaking from lysed cells

**Figure 6:**
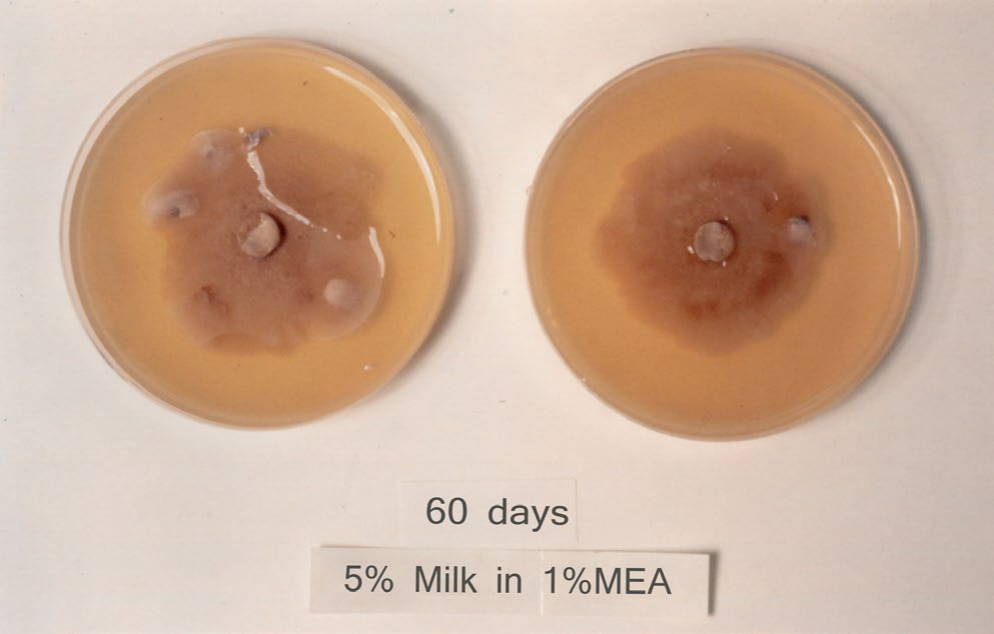
Milk malt extract agar, a medium which does support hyphal growth of *Chlorophyllum rhacodes*, shows clear zones in advance of the colonies, confirming exogenous protease production

*Chlorophyllum rhacodes* was found to produce exogenous proteases, which cleared milk agars (Figures 5 and 6).

### 3.3 Potential for consumption of *C. rhacodes* by *F. lugubris*

**Figure 7:**
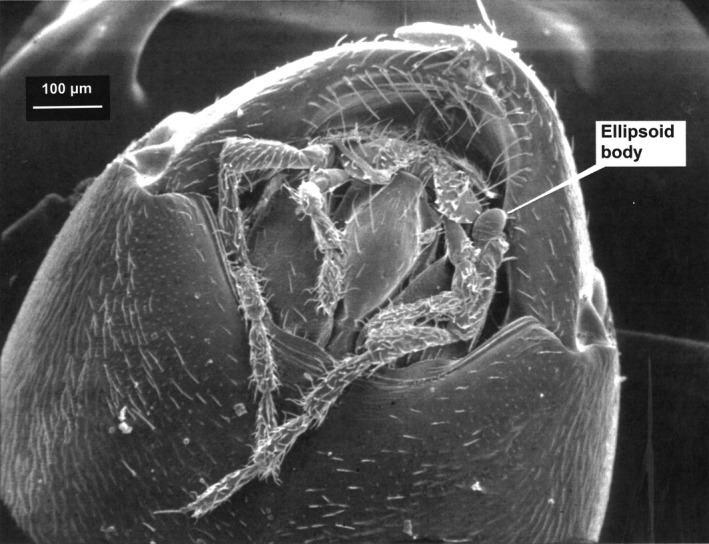
The mandibles of *Formica lugubris* viewed from below (ventral aspect). Note their long, pointed shape and widely spaced setae. The ellipsoid body measures approximately 40 µm (long axis). This ant's mouth measures more than 50 µm across

Figure 7 shows the ventral view of head and mouthparts. To the right of the mouth is an ellipsoid body, which is about four times the size of a *C. rhacodes* spore.

The mandibles are long and sharp; there are setae on the outer edge, typical of those used primarily for piercing exoskeletons. There are no obvious adaptations for mycophagy, although due to the size of spores and abundance of mycelium, it is likely that *C. rhacodes* could be consumed incidentally. Spores identical to those taken directly from *C. rhacodes* gills were found in fecal pellets of ants from the nests.

## 4 DISCUSSION

The fruiting length of the season contrasts with that limited to Autumn, as recorded by Pacioni (1985). Fruiting success appeared to be linked, spatially and temporally, to ant activity. *Formica lugubris* aggressively defends its territory (Elton, 1932; Savolainen, Vespsalainen, & Wuorenrinne, 1989) and are omnivorous, consuming largely honeydew, invertebrate (predated and scavenged), and plant material (Cherix & Bourne, 1980; Dumpert 1978; Fowler & Macgarvin, 1985;Laine & Niemelä, 1980; Robinson et al., 2008). These habits may protect the fruit‐bodies from ground attack by, for example, slugs and woodlice, but these activities are seasonal. The reduction in ant activity as a result of colder weather in late autumn and winter may be cause of the protracted period of successful fruiting to finally cease.

Sagara (1992) noted a physical association between the tree roots and dense, white mycelium and fruit‐body primordia initiation. This was again noted. However, in this longer observation, fruit‐bodies were found in two distribution patterns, linked to specific patterns of tree root invasion of the nests. The spatial link between roots and fruit‐body initiation suggests a chemical interaction in the rhizosphere. Indeed, the presence of ectomycorrhizal features on the roots suggested an intimate interaction. However, *C. rhacodes* was not shown to form the same extent of ectomycorrhiza using appropriately modified, standard laboratory technique, although there were traces of a weak Hartig net. However, this was not as extensively developed as the ectomycorrhiza in the nests, which, therefore, could not be attributed to *C. rhacodes*. It can be concluded that *C. rhacodes* was simply a major component of the rhizosphere, deriving its resources from sources other than a full physiological mycorrhizal association, unless there was an artifact caused by the complex community interactions of this specific rhizosphere (examples discussed below).

The facilitation of *C. rhacodes* fruiting in the conditions provided by long‐term *F. lugubris* nests, which are invaded by tree roots, suggests a possible connection between *C. rhacodes* and established woodland communities. While nest mound material is frequently cycled throughout the mound structure, the material in the highly branched root network is effectively trapped. This physical stability allows for growth of *C. rhacodes* among the litter, which is probably enriched by root exudates. Some unknown factors in the association between the fungus, roots, and ants lead to the extensive bifurcation of the tree roots (concomitant with an ectomycorrhizal symbiosis).

### 4.1 Relationship with resources provided by *F. lugubris*

Nests are composed of litter, and the frequent redistribution of the material maintains an environment suited to the ants and avoids rapid decomposition. Saprotrophic basidiomycetes are all believed to be capable of decomposing cellulose, hemicellulose, and lignin in litter (Valášková *et al*. 2007 *cited in* Žižka & Gabriel, 2008). The lepiotaceous fungi (including *C. rhacodes*) are rarely strong wood decomposers (outside the tropics) and normally utilize fast‐decomposing litter (Vellinga, 2004). Another of this group, *Leucocoprinus gongylophorus*, associated with attine ants, derives polysaccharides from sources other than cellulose (Bacci et al., 1995). The distribution of visually traceable *C. rhacodes* mycelium was associated with roots and insect cadavers, suggesting alternative sources of nutrition.

The large number of insect cadavers found in the rhizosphere areas of the nest mound presents a source of fatty acids and proteins. While there was no observed evidence of lipolytic activity, *C. rhacodes* was found to produce exogenous proteases and was found to be readily capable of hydrolysing white chitin.

Although there is a universal chitin for arthropods, annelids, and molluscs (Pearson, Marchessault, & Liang, 1960), the fact that a fungus can hydrolyze unpigmented, purified chitin does not necessarily mean that it can break down the dark chitin found in most insects. The exoskeleton has, essentially, two layers: the epicuticle and the procuticle (Hepburn & Joffe, 1976; Wigglesworth, 1984). The procuticle consists primarily of a protein‐chitin complex, in which chitin can account for 60% of the dry weight. It comprises two fundamental layers: the exocuticle and the endocuticle (Mordue, Goldsworthy, Brady, & Blaney, 1980). The endocuticle is chemically available and can be reabsorbed and reformed by the insect during life. It is a nutritional source for parasitic, or in a cadaver, saprotrophic fungi. To produce the tough and inelastic properties, exocuticular proteins are sclerotinized (tanned), *that is* adjacent proteins chains are cross‐linked by O‐quinones. Assuming that the binding quinones are more available during decomposition of the exocuticle, then it is relevant that *C. rhacodes* is possibly unique in producing high levels of quinone‐dependent sugar oxidoreductase, pyranose dehydrogenase (PDH), quite different from the PDH found in numerous white‐rot, saprotrophic fungi (Volc, Kubátová, Daniel, Sedmera, & Haltrich, 2001).

Although in vitro, *C. rhacodes* was found not to be able to degrade fresh, whole exoskeleton, the postmortem decomposition of this complex of materials does make chitin chemically available. It may be presumed that it is then available to the surrounding *C. rhacodes* hyphae in the accumulated cadavers within the nest.

### 4.2 Potential for mycophagy

The mandibles of *F. lugubris* are long and sharp; there are setae on the outer edge, but they are more distantly spaced than are normally found in spore combs (see Lawrence, 1987). These mandibles are typical of those used primarily for piercing exoskeletons, with no obvious adaptations for mycophagy. However, it is likely that *C. rhacodes* spores and hyphae could be consumed quite incidentally. Prey cadavers found in the rhizosphere areas of the nest mounds were covered in fungal mycelium, often close to fruit‐body initiation, and are likely to be decomposed by *C. rhacodes*. It can be assumed that if ants feed near these areas of *C. rhacodes* growth, inadvertent consumption of spores, and concentrated masses of hyphae is a likely occurrence, but only if ants return to those cadavers for nutritional purposes, of course. Although no hyphal remnants would be recognizable, spores exactly matching those of *C. rhacodes* were found in dissected ant fecal pellets. That they were whole and recognizable suggests that there was no, or minimal digestion, however.

### 4.3 Properties, stability, and function of nests of *Formica lugubris*

Social community structure is a feature observed in, but not exclusive to, the Insecta. Among the insects, levels of highly developed eusociality are varied, but probably most familiar in the orders: Dictyoptera, which includes termites, and Hymenoptera, which includes ants (Feldhaar, 2014; Krishna & Weesner 1970a,1970b; Nowak, Tarnita & Wilson, 2010; Robinson, 2008). Throughout the world, ants have evolved societies exploiting nearly every conceivable way of living in the terrestrial environment. In the tropics, colonies may contain hundreds of thousands of individuals. In temperate regions, colonies are normally less populous, but this social organization is no less impressive. In terms of numbers and omnipresence, ants must be regarded as one of the most successful groups of animals. This may be due to the division of labor (resulting from polyethism) expressed in reproduction, food collection and feeding, and in defense (Dall, Bell, Bolnick, & Ratnieks, 2012; Feldhaar, 2014; Nowak et al., 2010; Robinson, 2008). Task allocation is often ensured by the development of a system of castes, which are so differentiated that none can survive without the others (Beattie 1985; Brian, 1977; Dumpert, 1978), with subtle variations with evolutionary potential, dependent on individuals' preferences (see Dall et al., 2012).

*Formica* *lugubris* builds polygynous, sometimes polydomous, colonies ranging from thousands to tens of thousands of workers and the size of the nest is partly dependent on its age (Breen, 1979a; Gyllenstrand & Seppä, 2003; Robinson, 2008; Robinson et al., 2008). In some situations, *F. lugubris* was regarded as primarily insectivorous (Cherix & Bourne, 1980; Finnegan, 1975; Gösswald, 1958; Laine & Niemelä, 1980; McNeil, 1977), although ants are notoriously omnivorous (Dumpert 1978; Robinson et al., 2008). Honeydew (secretions from aphids) is an important food source for many ants in temperate biomes. In season, and particularly in broadleaved woodlands, it has been estimated that 75% of *F. lugubris* foragers carry honeydew back to the nest, while most other food is insect prey, caught or scavenged (Breen, 1979b; Sudd, 1983). Evidence suggests that seasonal changes in spruce needle sap nutrients have a decisive influence on vital activities and population size of some aphids (Day, Armour, & Doherty, 2004). In a monoculture plantation, as is Highlow Wood, seasonal variations will have greatest effect. It is likely that in periods of low aphid numbers, ants in conifer plantations will be nutritionally more dependent on fresh or scavenged insect cadavers.

Nests comprise a built mound element as well as excavations in the ground beneath the mound. There are tunnels and galleries in both super‐ and subterranean elements. Entrances to the mound appear to be transient features, possibly associated with ventilation requirements. The mound can reach just over a meter in height above ground level. Typically, these mounds are constructed near the base of a tree and solely of the leaves and small twigs from nearby trees (or uniformly cut lengths of ground flora where trees are less abundant). In the sessile oak woodlands of South Yorkshire, nest mounds commonly contain a substantial proportion of primordial acorns still attached to peduncle and short twig (author's observation). In the pine plantations, such as Highlow Wood, the nest mounds are largely pine needles. As per usual, nests are sited most frequently where they benefit from easterly or southerly sunshine (as Kilpeläinen et al., 2008; Robinson et al., 2008; Sudd, Douglas, Gaynard, Murray, & Stockdale, 1977).

Ants may maintain a nest location for as long as 20 years. During occupation, nest material is constantly cycled from inside outwards. All materials deemed alien are diligently removed by the ants, where possible (Elton, 1932); this includes seedlings that germinate in the nest and soft plant tissue, which falls onto the nest surface. This dynamic system ensures a mound of consistently uniform composition, avoiding the problems of collapse, temperature increase and water‐logging associated with the rotting of heaped vegetable matter (Kilpeläinen et al., 2008). In regulating their immediate environment, the ants are providing conditions that may impact on resource utilization by other organisms.

### 4.4 Fungus – ant mutualism; evolution of a diet‐based symbiosis

There are many ways in which ants manipulate other organisms and vice versa. It has long been believed that ant‐fungus mutualisms are probably the result of incidental, spatial coexistance, where the activities of both organisms happened to produce enhanced benefits to each other and which lead to ever more developed association (Emery, 1899). An extraordinary example of obligatory mutualism, commanding a great deal of mycological attention, is exhibited by the tribe Attini (Formicidae: Myrmicinae). These ants cultivate saprotrophic fungi in special areas within their nests. Most of these ant species grow the fungi on collected feces or senesced plant material. Those genera considered to be more evolutionarily advanced, *Atta* and *Acromyrmex*, provide their fungi with fresh leaves or flowers. In tropical rainforests, this harvest may account for 17% of the total leaf production. The ants eat parts of the fungus, which is cultivated on prepared foliage substrate. Preparation involves the application of acidic anal excretions containing detoxifying and digestive enzymes, which can only be produced intracellularly by the fungus. These fungal enzymes are liberated during mastication by the ant (Martin, 1984; Slanski & Rodriguez 1987). Constant inoculation of new substrate by the ants, together with the antibiotics in the fecal‐enzyme cocktail may maintain the overwhelming state of monoculture in the fungus gardens (Martin, 1970; Powell & Stradling, 1986; Schilnecht & Koob, 1970, 1971; Weber, 1955). As a result of facilitating the fungus nutrition, the ant derives food in greater mass and closer proximity than would be available, otherwise. For example, the garden fungus *Leucocoprinus gongylophorus* contains glucose, which it derives from cellulose in leaves (Bacci et al., 1995). The ant consumes the glucose‐rich fungus, thereby receiving glucose from a plentiful source for which they have no enzymes to derive it directly. Further highlighting the specialized microecosystem of the nest, the fungus garden parasite *Escovopsis* sp. (Ascomycotina) is thought to be controlled by a bacterium of the genus *Streptomyces* (Actinomyces) (Currie, Scott, Summerbell, & Malloch, 1999).

It is quite possible that the fungi cultivated by some ant species do not fruit anywhere other than on abandoned nests, where tending has ceased and chemical regime has altered. Laboratory tests demonstrated that species of *Phallus*, *Agaricus,* and *Lepiota* have been accepted by ants (Weber, 1938). In natural nests, the favoured species are probably of *Leucocoprinus* and *Leucoagaricus* lineage (genera of the Lepiotacea) where fruit‐bodies possess free gills and basidiospores with germ pores. DNA analyses show that *C. rhacodes* is more closely related to the *Leucoagaricus* clade than the species type of its former genus, *Macrolepiota*, hence, the reclassification as *Chlorophyllum rhacodes* (Ge et al., 2018; Vellinga, de Kok, & Bruns, 2003). As the taxonomic status of the attine‐farmed fungus (or fungi) is still doubtful, the generic name *Attamyces* has been contrived to suggest a comparable domesticated lifestyle with that of the termite associates *Termitomyces* (Cherrett, Powell, & Stradling, 1987). The “higher” termites (Termitidae: Macrotermitinae) provide an old‐world ecological equivalent to the attines (Emerson, 1955; Krishna & Weesner 1970a,1970b; Lee & Wood, 1971). The slow‐growing Termitomyces is provided with a finely divided substratum of termite feces, chemical and biologic constituents of which prevent the development of certain microorganisms and parasites, rendering the garden (comb) community dominated by Termitomyces fungus (Zoberi & Grace, 1990).

Control of detrimental fungal insurgents is as important to all ants, as it is to the fungus cultivators. No ant habitats are ever likely to be fungus‐free, but the majority of interactions between the ants and the fungi are usually casual. Notable exceptions are entomogenous fungi, against which (in addition to a hard, waxy cuticle and a complex laminate exoskeleton) ants utilize their many chemical means of protection. A comprehensive range of fungistats and antigerminants are produced—the reason why so few ants are pollinators (Beatie, 1982; Beatie, Turnbull, Knox, & Williams, 1984; Iwanami & Iwadare, 1978, 1979; Iwanami, Iwadare, Okada, & Iwamatsu, 1979) and relatively few are responsible for the dissemination of seeds (myrmecochory) (Beattie, 1985; Brand, Page, Lindner, & Markovetz, 1989; Culver & Beattie, 1978; Dumpert, 1978; Hori, 1976; Webster, 1980) (but see Berg, 1975; Giladi, 2006; Kinkaid, 1963 for exceptions). Mandibular, anal and metathoracic (metapleural) gland secretions, the latter playing a part in colony recognition (Brown, 1968; Maschwizt, 1974), are responsible for many antibiotic effects. The reservoirs of the metathoracic glands, into which several other glands open, cannot be closed so that the antibiotic secretions are constantly distributed over the body surface (Maschwizt, 1974). Nevertheless, active fungi are found in all ants' nests. Wherever ants gather and concentrate their food or excretions, and wherever their environmental manipulations increase the availability of resources, certain fungi will colonize there. Indeed, the most acceptable theory for the origin of fungus cultivation is that it evolved from a situation where a fungus grew abundantly on ant feces accumulated in a gallery store (Dumpert 1978). Thus, there is potential in most ant situations, bearing in mind that all ants are to some extent omnivorous, for the evolution of some kind of diet‐based symbiosis.

### 4.5 Effects of diet on insect behavior and morphology

Insect diet has profound effects on physiological development and behavior. Differences between castes of social insects are phenotypical, that is they are the result of switching on or off genes that are present in all individuals of a species. Switching is the result of specific cues received at precise stages during development of the larvae or instars (Evans & Wheeler, 2001). In most insects exhibiting a caste system, an obvious physiological and behavioral extreme is between the worker and the queen. The phenotypical distinction (polyethism) is caused by a different balance of nutrients fed to the larvae. In honey bees (*Apis mellifera*), there are three distinct food preparations: worker jelly, young worker jelly, and royal jelly. These preparations are different mixtures of the same mandibular gland secretions, hypopharageal gland secretions, and crop contents. After only three days of development, the change in balance of these preparations in a larva's diet will determine its development to sexual maturity or suppression and to physical and behavioral distinctions. While some of the hormones involved are derived from the nurse workers' metabolism, some of the active chemicals are from crop contents (Wheeler, Buck, & Evans, 2006), thus are foraged food constituents. This highlights the importance of major changes or additions to the insects' diet.

### 4.6 Endocrine disruption, behavior changes and mutualism

Hormonal disruption is one factor in a range of possible mechanisms in the alteration of insect behavior during infection by fungal parasites (Adamo, 2013; Andersen et al., 2009; Hughes et al., 2011; Hughes, Brodeur, & Thomas, 2012; Pontoppidan, Himaman, Hywel‐Jones, Boomsma, & Hughes, 2009). Such influence, which directly benefits the parasite growth and dispersal results from complex influences on the insects' diurnal rhythms and so must be the result of evolutionary development (de Bekker, Merrow, & Hughes, 2014).

As consumption of specific, hormonally active foods and exposure to endocrine disruptive metabolites is a common feature of insect life, it is particularly conceivable that this is the origin of insect‐fungus mutualisms. Mutualistic symbioses can be regarded as highly evolved parasitism (where the in‐host phase has been extended by causing no harm, or even causing benefit to the host) (de Bary, 1887, *cited in* Smith & Read, 1997). It also makes sense that greater specialization in diet can lead to increased nutritional efficiency, if all other factors remain the same (Bernays, 2001), which suggests a possible evolutionary pressure toward mutualisms.

### 4.7 Synthesis

The association between fruiting and rich, stable, humic substrata suggests that *C. rhacodes* might serve as an indicator of historic woodland and unimproved woodland pasture.

Based on this investigation, an argument has been made supporting the theoretical process of the development of the fungus‐farming mutualisms, most clearly exhibited by the attine ants. It demonstrates that the necessary stages of facultative development does happen and that complex ecological community interactions are worthy of further research.

## 5 CONCLUSIONS

Through the actions of the *F. lugubris* colony, physical and chemical conditions in the nests are suitable for proliferation of *C. rhacodes* mycelium, including fruit‐body initiation and protection from mycophagy.

Conditions that develop in the rhizosphere within the ant nest mounds (probably as a result of plant, ant and presumed microbial activities) appeared to stimulate fruiting of *C. rhacodes*, suggesting a facultative association.

The facilitation of *C. rhacodes* fruiting in the conditions provided by long‐established nests, suggests a possible connection between *C. rhacodes* and woodland communities. A link between *C. rhacodes* distribution and historic woodland pasture is worth investigating.

*Chlorophyllum rhacodes* is theoretically capable of utilizing degraded insect exoskeletons as a resource and was found present in the nest stores of insect cadavers. In terms of chitin metabolism, *C. rhacodes* presents no predatory threat to living insects, but is able to utilize chitin and some proteins, released by decomposition of cadavers.

*Formica lugubris* is likely to consume quantities of *C. rhacodes* mycelium along with their stored food supplies.

The saprotrophic nature of *C. rhacodes* will release sugars from pine‐needle derived cellulose and if consumed by *F. lugubris*, will enhance its nutrition, hinting at a facultative association.

As insects are prone to large‐scale morphological and behavioral alterations as a result of different diets, an increase in specific mycelium in their food presents such an opportunity. Among all the possible outcomes, one might be a tendency to select for behavior that maintains or further encourages the presence of *C. rhacodes* thereby developing into a more obvious mutualism, by the mechanism postulated over one hundred years ago (op. cit.).

Inherent in all these observations is the possibility of the evolution of an obligate ant‐fungus mutualism. Although the most strongly developed fungus‐farming relationships are only known in tropical biomes, the warming temperate climate presents opportunities for changing distributions and interactions of potential symbionts.

## AUTHOR'S CONTRIBUTION

Douglas Fraser is the sole author of this work. Where results, theories or concepts have been drawn from other authors, these have been properly acknowledged and referenced.

## Data Availability

This article does not contain any additional data.
